# Neonatal admission after lithium use in pregnant women with bipolar disorders: a retrospective cohort study

**DOI:** 10.1186/s40345-023-00306-7

**Published:** 2023-07-14

**Authors:** Noralie N. Schonewille, Pleun A. Terpstra, Maria E. N. van den Heuvel, Maria G. Van Pampus, Odile A. van den Heuvel, Birit F. P. Broekman

**Affiliations:** 1 Department of Psychiatry and Medical Psychology, OLVG, Oosterpark 9, Amsterdam, The Netherlands; 2 Department of Psychiatry, Amsterdam UMC, Vrije Universiteit Amsterdam, Boelelaan 1117, 1081 HV Amsterdam, The Netherlands; 3 Amsterdam Public Health Program, Amsterdam, The Netherlands; 4 Department of Paediatrics, OLVG, Oosterpark 9, Amsterdam, The Netherlands; 5 Department of Obstetrics and Gynaecology, OLVG, Oosterpark 9, Amsterdam, The Netherlands; 6 Department of Psychiatry, Department of Anatomy & Neurosciences, Amsterdam UMC, Vrije Universiteit Amsterdam, Boelelaan 1117, Amsterdam, The Netherlands; 7 Amsterdam Neuroscience, Compulsivity, Impulsivity and Attention Program, Amsterdam, The Netherlands

**Keywords:** Lithium, Bipolar disorder, Adverse neonatal outcomes, Psychiatry, Pregnancy

## Abstract

**Background:**

Lithium is the preferred treatment for pregnant women with bipolar disorders (BD), as it is most effective in preventing postpartum relapse. Although it has been prescribed during pregnancy for decades, the safety for neonates and obstetric outcomes are a topic of ongoing scientific debate as previous research has yielded contradicting outcomes. Our study aims to compare (re)admission rates and reasons for admissions in neonates born to women with bipolar disorders (BD) with and without lithium exposure.

**Methods:**

A retrospective observational cohort study was conducted in a Dutch secondary hospital (two locations in Amsterdam). Women with BD who gave birth after a singleton pregnancy between January 2011 and March 2021 and their neonates were included. Outcomes were obtained by medical chart review of mothers and neonates and compared between neonates with and without lithium exposure. The primary outcome was admission to a neonatal ward with monitoring, preterm birth, small for gestational age (SGA), 5-minute Apgar scores, neonatal asphyxia, and readmission ≤ 28 days.

**Results:**

We included 93 women with BD, who gave birth to 117 live-born neonates: 42 (36%) exposed and 75 (64%) non-exposed to lithium. There were no significant differences in neonatal admission with monitoring (16.7 vs. 20.0%, p = 0.844). Additionally, preterm birth (7.1 vs. 5.3%), SGA (0.0 vs. 8.0%), 5-minute Apgar scores (means 9.50 vs. 9.51), neonatal asphyxia (4.8 vs. 2.7%) and readmission (4.8 vs. 5.3%) were comparable. Overall, 18.8% of BD offspring was admitted. Women with BD had high rates of caesarean section (﻿29.1%), gestational diabetes (12.8%) and hypertensive disorders of pregnancy (8.5%).

**Conclusions:**

In a sample of neonates all born to women with BD using various other psychotropic drugs, exposure to lithium was not associated with greater risk of neonatal admission to a ward with monitoring compared to non-exposure to lithium, questioning the necessity for special measures after lithium exposure. However, offspring of women with BD was admitted regularly and women with BD have high obstetric risk which require clinical and scientific attention.

## Background

Lithium is the preferred treatment for pregnant women with bipolar disorders (BD), as it is most effective in preventing postpartum relapse (Wesseloo et al. 2016; Gilden et al. 2021). Although it has been prescribed during pregnancy for decades, the safety for neonates and obstetric outcomes are a topic of ongoing scientific debate (Hastie et al. 2021). Previous research has yielded contradicting outcomes. Various cohort studies and systematic reviews (with meta-analyses) have found no increased risk in obstetric outcomes such as hypertensive disorders of pregnancy and gestational diabetes in women with BD with versus without lithium use (Poels et al. 2018; Munk-Olsen et al. 2018; Bodén et al. 2012; Fornaro et al. 2020; Sagué-Vilavella et al. 2022). Yet, some studies have suggested a small increased risk of spontaneous abortion, preterm birth, and increased birthweight after maternal lithium use (Hastie et al. 2021; Poels et al. 2021). Others described an association of BD with adverse obstetric and pregnancy outcomes such as increased risk of caesarean section, small- or large for gestational age neonates and preeclampsia (Vigod et al. 2014; Rusner et al. 2016). Moreover, previous research has reported an association between lithium and congenital malformations (Hastie et al. 2021; Fornaro et al. 2020; Patorno et al. 2017), lower Apgar scores (Sagué-Vilavella et al. 2022; Newport et al. 2005) and neonatal readmission within 28 days postpartum (Munk-Olsen et al. 2018). Although reasons for admission of lithium-exposed neonates were not available, the authors suggested that admissions were the result of increased vigilance towards neonates exposed to lithium, neonatal withdrawal syndrome and vulnerability of neonates due to impaired maternal mental health.

Most guidelines and hospitals preventatively admit neonates to monitor their condition postpartum (Vigod et al. 2014; Rusner et al. 2016). However, admission to a neonatal ward with monitoring may also have adverse consequences. It can lead to increased feelings of parental stress, and a negative effect on early mother-infant attachment, which is especially important for patients who are already vulnerable for mental health disorders (Obeidat et al. 2009; Al Maghaireh et al. 2016). Preferably, admission to a neonatal ward with monitoring for lithium-exposed neonates should be evidence-based. As also mental health disorders may impact obstetric outcomes it is important to differentiate lithium-related adverse outcomes from adverse outcomes related to the mental disorder. The aim of the current study is to validate previous findings on neonatal outcomes after lithium exposure by comparing (re)admission rates in neonates born to women with BD with versus without lithium exposure. Moreover, we aim to elucidate the reasons for admissions in neonates born to women with BD.

## Methods

### Participants and eligibility criteria

Methods were reported according to the STROBE checklist (https://www.equator-network.org/reporting-guidelines/strobe/) for reporting observational cohort studies. The retrospective cohort consisted of a convenience sample of neonates of singleton pregnant women, ≥ 18 years, with BD diagnosed by a psychiatrist before pregnancy, or clear symptoms of BD before pregnancy and confirmed diagnosis (by a psychiatrist) postpartum. Participants gave birth to a liveborn neonate between January 2011 and March 2021 at OLVG hospital (a large secondary care hospital in Amsterdam, the Netherlands), or had their child admitted directly postpartum after home delivery due to complications or as per protocol. In the Netherlands, obstetric care is divided between community midwifes (primary care), obstetrician-gynaecologists (secondary care) and academic referral centres (tertiary care). When lithium was used at *any* point throughout the pregnancy, neonates were included in the lithium-exposed group (including women who started lithium after the first trimester (n = 3)), other neonates were included in the non-lithium exposed group. We excluded women with an uncertain diagnosis of BD, twin pregnancies, or records with missing information on maternal and neonatal outcomes. All records were hand searched by one researcher and discussed with a second or third researcher if necessary to prevent misclassification of outcomes.

The Medical Research Involving Human Subjects Act was not applicable for this study. The study was approved by the Advisory Committee Scientific Research at OLVG hospital who granted exemption for written informed consent because of the large number of records to search.

### Study variables and definition of outcomes

We primarily investigated the number of, and reasons for admissions to the neonatal ward with monitoring (level 2 care). In OLVG hospital, neonates born to women with BD are observed for minimally 24 h while roomed in with their mothers on the maternity ward (level 1 care, which has no opportunity for continuous monitoring of vital parameters) and only admitted to the neonatal ward with monitoring when indicated by the paediatrician. In other Dutch hospitals, admission to a neonatal ward with monitoring of vital parameters for at least 24–48 h is generally the norm. Data on all other adverse outcomes in neonates admitted within 28 days postpartum was collected from obstetric and neonatal patient files. Data on the mothers’ sociodemographic characteristics, medication use (including lithium dosage of pregnant women), and neonatal outcomes were extracted from patient files. Prematurity was defined as delivery before 37 weeks of gestation, large for gestational age as weight above the 97th percentile, small for gestational age under the 10th percentile (Hoftiezer et al. 2019) and maternal obesity as a body mass index > 30 kg/m^2^.

### Statistical analysis

Statistical analyses were performed using R Studio version 4.0.4 (www.rstudio.com). For normally distributed continuous baseline characteristics, means and standard deviations were calculated with T-tests to assess differences between groups. Categorical and dichotomous variables were presented with numbers and percentages per category and Chi^2^ tests were performed to assess differences. The primary outcome (re)admission to a neonatal ward with monitoring was compared between groups using a Chi^2^ test and additional logistic regression analysis was performed to adjust for psychotropic medications other than lithium as a dichotomous variable. Odds ratio (OR) with a 95% confidence interval and p-values were reported.

## Results

Out of 32,705 birth registration records, we identified 970 records of women with the term ‘bipolar disorder’ in their electronic files, of which 119 women met inclusion criteria and were evaluated in-depth. Subsequently, 26 women were excluded because of unclear diagnosis of BD (n = 3) and missing data on maternal, delivery and neonatal outcomes (n = 23). A total of 117 liveborn neonates were included, born to 93 women. See Table 1 for maternal and pregnancy characteristics.

**Table 1:** Descriptive characteristics of study sample

	Total (n = 117)	Non-lithium exposed group (n = 75)	Lithium-exposed group (n = 42)	P-value
Maternal and pregnancy characteristics				
Maternal age at delivery, years mean (SD)	34.31 (4.24)	34.79 (4.21)	33.45 (4.20)	0.102
Multipara, n (%)	51 (43.6)	28 (37.3)	23 (54.5)	0.198
Intoxication*, n (%)	23 (19.7) Missing 3	17 (22.7) Missing 3	6 (14.3)	0.340
Smoking, n (%)				0.698
No	95 (81.2)	59 (78.7)	36 (85.7)	
Yes, all trimesters	13 (11.1)	9 (12.0)	4 (9.5)	
Yes, only in first trimester	8 (6.9)	6 (8.0)	2 (4.8)	
Lithium exposure				
During pregnancy at any point, n (%)	42	NA		
In third trimester, n (%)	41	NA		
Dosage, mean mg/day (range)	1051.28 (400–2400) Missing 5	NA		
Psychotropic medication other than lithium, n (%)	66 (56.4)	44 (58.6)	23 (54.8)	0.940
Typical antipsychotics	8 (6.8)	4 (5.3)	4 (9.5)	NA
Atypical antipsychotics	39 (33.3)	26 (34.7)	13 (31.0)	0.838
SNRI	6 (5.1)	5 (6.7)	1 (2.4)	NA
SSRI	12 (10.3)	6 (8.0)	6 (14.3)	0.4488
TCA	1 (0.9)	0 (0.0)	1 (2.4)	NA
TeCA	1 (0.9)	1 (1.3)	0 (0.0)	NA
Benzodiazepines	7 (6.0)	5 (6.7)	2 (4.8)	NA
Atypical antidepressants	2 (1.7)	1 (1.3)	1 (2.4)	NA
Anticonvulsants	15 (12.8)	11 (14.7)	4 (9.5)	NA
Type of bipolar disease, n (%)				0.012**
I	46 (39.3)	21 (28.0)	25 (59.5)	
II	54 (46.2)	39 (52.0)	15 (37.5)	
Rapid cycling	4 (3.4)	2 (2.7)	2 (4.8)	
Not otherwise specified	2 (1.7)	2 (2.7)	0 (0.0)	
Planned pregnancy, n (%)	70 (60.0) Missing 22	42 (56.0) Missing 16	28 (66.7) Missing 6	0.640
Obesity, n (%)	25 (21.4) Missing 40	12 (16.0) Missing 28	13 (31.0) Missing 12	0.168
Pregnancy outcomes				
Gestational diabetes mellitus***, n (%)	15 (12.8)	9 (12.0)	6 (14.3)	0.947
Hypertensive disorders of pregnancy,*** n (%)				0.218
No	107 (91.5)	71 (94.7)	36 (85.7)	
Pregnancy induced hypertension	4 (3.4)	2 (2.7)	2 (4.8)	
Preeclampsia	6 (5.1)	2 (2.7)	4 (9.5)	
Polyhydramnios, n (%)	7 (6.0) Missing 40	2 (2.7) Missing 30	5 (11.9) Missing 10	0.201
Caesarean section, n (%)	34 (29.1)	18 (24.0)	16 (38.1)	0.162
Of which unplanned n (% of total caesarean sections per group)	22 (64.7)	13 (72.2)	9 (56.3)	0.540

*NA* not applicable, *GAD* gestational age at delivery, *n* number, *IQR* interquartile range, *SD* standard deviation.

*Alcohol/smoking/recreational drugs, **p < 0.05, ***According to local protocols

Two women were diagnosed with BD in the postpartum period, 91 women were diagnosed before or during pregnancy. Some women had several pregnancies. Forty-two neonates were exposed to lithium during pregnancy and 75 were not. Thirty-nine lithium-exposed neonates (93%) were already exposed to lithium from the first trimester onwards. Of the 42 women in the lithium exposed group, 41 women used lithium during pregnancy until delivery and continued lithium use in the first 28 days postpartum. One woman caesed lithium in the first trimester of pregnancy. Type of lithium was unknown in five women and the other 37 women used a form of lithium carbonate with dosages between 400 and 2400 mg a day (see Table 1). Psychotropic medication other than lithium was used frequently in both groups (54.8% in lithium-exposed and 58.6% in non-lithium exposed neonates). Nine out of 42 lithium-exposed neonates were also exposed to antipsychotics (21.4%), four to serotonergic antidepressants (9.5%) and eight neonates to a combination of psychotropic medication (19.0%). In the non-lithium exposed neonates, antipsychotics (as monotherapy) were most frequently reported during pregnancy (25.3%), followed by serotonergic antidepressants (as monotherapy) in four cases (5.3%) and 13 neonates were exposed to a combination of psychotropic medication (17.3%). Type of BD differed between groups (with more type-I in the lithium-exposed group), all other characteristics were comparable between mothers.

### Admission to a neonatal ward with monitoring and adverse neonatal outcomes up to 28 days postpartum

Overall admission rate to a neonatal ward with monitoring was 19%, with a median duration of 3 days, see Table 2.

**Table 2:** Neonatal outcomes according to lithium exposure

	Total (n = 117)	Non-lithium exposed group (n = 75)	Lithium-exposed group (n = 42)	P-value
Neonatal outcomes				
Sex, female, n (%)	62 (53.0)	44 (58.7)	18 (42.0)	0147
Gestational age, mean (SD)	276.23 (11.10)	276.55 (11.27)	275.67 (10.92)	0.283
Gestational age, weeks + days (SD)	39 + 4 (1 + 5)	39 + 5 (1 + 6)	39 + 4 (1 + 5)	0.283
Preterm birth, n (%)	7 (6.0)	4 (5.3)	3 (7.1)	NA
Birth weight in grams, mean (SD)	3445.16 (492.94)	3375.36 (481.14)	3569.81 (494.80)	0.040*
Percentile weight, mean (SD)	51.25 (28.58)	46.72 (27.55)	59.33 (28.92)	0.021*
Large for gestational age, n (%)	12 (10.3)	6 (8.0)	6 (14.3)	0.449
Small for gestational age, n (%)	6 (5.1)	6 (8.0)	0 (0.0)	NA
Apgar scores				
Apgar score 1 min, mean (SD)	7.75 (2.21)	7.71 (2.32)	7.83 (2.01)	7.758
Apgar score 5 min, mean (SD)	9.50 (1.13)	9.51 (1.26)	9.50 (0.89)	0.973
Neonatal asphyxia**, n (%)	4 (3.4)	2 (2.7)	2 (4.8)	NA
Admission to neonatal ward with monitoring, n (%)	22 (18.8)	15 (20.0)	7 (16.7)	0.844
Duration admission to care unit in days, median (IQR)	3 (5.00)	5 (5.00)	2 (0.75)	NA
Congenital malformations, n (%)	8 (6.9)	4 (5.3)	4 (9.5)	NA
Readmission ≤ 28 days postpartum	6 (5.1)	4 (5.3)	2 (4.8)	NA

*n* number, *SD* standard deviation, *IQR* interquartile range, *NA* not applicable

Chi^2^ tests were not applicable if value in cell < 5

*p < 0.05.

** According to local protocol

Lithium-exposed neonates had a significantly higher birth weight (p = 0.040) and birth weight percentile (p = 0.021) than non-exposed neonates. There was no significant difference in admission rate to a neonatal ward with monitoring between lithium-exposed and non-lithium exposed neonates when estimated using a Chi^2^ test (OR 0.80, 95% CI 0.25–2.34, p = 0.844), a logistic regression analysis without any covariates (OR 0.80, 95% CI 0.28–2.09, p = 0.658) and after adjustment for ‘other psychotropic medication than lithium’ (OR 0.75, 95% CI 0.26–1.99, p = 0.671).

A posthoc sensitivity analysis was performed to assess neonatal admission for neonates exposed to lithium in the third trimester of pregnancy (n = 41) versus neonates not exposed to lithium in the third trimester of pregnancy (n = 76) (OR 0.84, 95% CI 0.26–2.45, p = 0.808 using a Chi^2^ test).

**Fig. 1:**
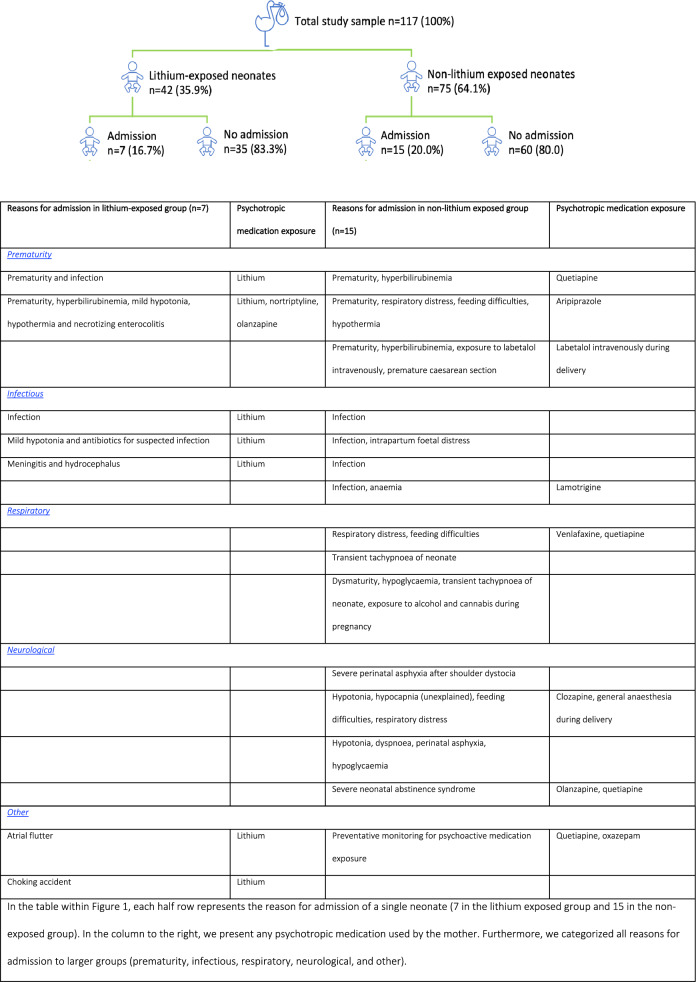
Neonatal outcomes according to lithium exposure during pregnancy

Figure 1 demonstrates reasons for admission to a neonatal ward with monitoring in both lithium-exposed and non-lithium exposed neonates, in addition to other psychotropic medication used in neonates with complications.

One serious adverse outcome possibly related to lithium exposure was observed: a case of atrium flutter that required adenosine treatment. Three lithium-exposed neonates had glandular hypospadias, as opposed to four congenital malformations in the non-lithium exposed neonates (congenital foot deviation, polydactyly, ventricular septal defect, and central congenital hypothyroidism). Two lithium-exposed neonates were readmitted to a neonatal ward with monitoring within 28 days postpartum (the neonate with atrial flutter for relocation from an academic centre and one neonate with progressive posthemorrhagic hydrocephalus) and four non-lithium exposed neonates were readmitted (one with congenital hypothyroidism and adrenal insufficiency, two with feeding difficulties, one after relocation because of shoulder dystocia with severe asphyxia).

## Discussion

### Main findings

There was no association between lithium exposure and admission to a neonatal ward with monitoring during the first 28 days after delivery (p = 0.844). One (1%) serious adverse outcome was possibly related to lithium exposure. One in five neonates born to women with BD, independent of lithium exposure, required admission to a neonatal ward with monitoring. Remarkably, pregnant women with BD had high obstetric vulnerability shown by high caesarean section, gestational diabetes, and hypertensive disorders. Our findings that lithium exposure is not associated with more frequent admissions to a neonatal ward with monitoring in a sample of women with BD only is in concordance with findings from a sensitivity analysis in the study of Munk-Olsen et al. (2018).

### Strengths and Limitations

Our focus on neonatal admissions to a ward with monitoring after lithium exposure in solely women with BD is novel. Many studies have focused primarily on congenital malformations or birth outcomes instead of the necessity of monitoring the neonate for 24 h on a neonatal ward, which is an important clinical outcome that affects parents and neonates. Moreover, inclusion of a control group consisting of neonates born to women who were diagnosed with BD decreased confounding bias. This is important, as mental disorders itself, including BD, have shown to increase the risk of pregnancy, obstetric and neonatal adverse outcomes (Bodén et al. 2012; Scrandis 2017). However, this study has potential limitations. First and foremost, analyses were based on a limited sample of neonates, leading to a lack of statistical power when discussing neonatal outcomes, and reason for (re)admission, with low prevalence. This is also applicable to the primary outcome neonatal admission after lithium exposure. Within this sample, lithium-exposed versus non-exposed neonates differed regarding type of BD, which we were unable to correct for due to the limited sample size. Moreover, as 93% of neonates were already exposed to lithium in the first trimester, we were unable to create different research groups to investigate possible differences in first, second or third trimester lithium use. Lithium levels of these women were monitored externally and could not be accessed. Due to the careful monitoring and information of lithium dosage, it is probably safe to assume lithium levels were within therapeutic range. However, other factors such as hydration status could impact lithium levels in neonates. In future studies it is advised to include lithium levels. Also, we found low numbers of women diagnosed with BD recorded in our hospital’s charts in this sample compared to the lifetime prevalence of BD (Merikangas et al. 2007). Possibly, women with BD were underrepresented or underdiagnosed in OLVG hospital. This would be worrisome, as women with severe mental illness deserve and require tailored obstetric care which is available at the specialised clinic for pregnancy and psychiatric vulnerability in OLVG hospital. Another noteworthy limitation is the common use of psychotropic medication other than lithium in both groups. Although we have adjusted for other psychotropic use in our logistic regression to understand the effect of lithium aside from other psychotropic drugs, overall the other psychotropic medicine (such as antipsychotics (typical and atypical), various types of antidepressants, benzodiazepines, and anticonvulsants could have influenced birth outcomes in both groups, and therefore admission to a neonatal ward with monitoring (Bodén et al. 2012). Given this influence of psychotropic medication, we have no information on a causal association between lithium exposure and neonatal admission to a ward with monitoring. On the other hand, the diversity in medication use will make our results more generalizable to the clinical population, as many patients with BD use multiple psychotropic medications. As lithium-exposed neonates were not standardly monitored, not all anomalies might have been discovered. This can be considered a strength rather than a limitation because neonates born after an uncomplicated pregnancy would also not be monitored. It can therefore be argued that potentially missed anomalies were clinically insignificant and would have skewed the results towards an overestimation of adverse outcomes in lithium-exposed neonates compared to the reference group.

### Interpretation

Similar admission rates between lithium and non-lithium exposed neonates seem to contradict previously described increased admissions to a neonatal ward with monitoring after lithium exposure due to floppy infant syndrome, cardiac arrhythmia, thyroid disorder, congenital malformations and Ebstein anomaly (Fornaro et al. 2020). However, our relatively high overall admission rate to a neonatal ward with monitoring of 19% may be related to the BD status of the mother more than to lithium exposure to the neonate. This is argued by comparable admission rates between lithium-exposed neonates and neonates born to mothers with BD in previous study samples (Munk-Olsen et al. 2018). Our findings showed lower absolute rates of (re)admission to a neonatal ward with monitoring in both groups compared to the previously described 27.5% in lithium-exposed neonates in the study of Munk-Olsen et al. (2018). As the reason for (re)admission is not described in the study of Munk-Olsen, we are unable to interpret this difference in (re)admission rate within the first 28 days of life. In our study, neonates with severe adverse outcomes were identified immediately postpartum in the delivery rooms or the maternity ward and not through preventative admission to a neonatal ward with monitoring. More importantly, none of the lithium-exposed neonates who were not initially admitted to a neonatal ward with monitoring, suffered any adverse outcomes within the first 28 days postpartum. For these neonates, rooming-in with the mother on a maternity ward without monitoring was safe and, according to earlier research, beneficial for parental mental health and parent-infant bonding (Obeidat et al. 2009; Al Maghaireh et al. 2016; Veenendaal et al. 2020a, b).

As for the possible lithium-related admission to the neonatal ward with monitoring, an atrial flutter was observed in a lithium-exposed neonate at 37 + 3 weeks’ gestation. A previous case was described in 1983 (Wilson et al. 1983), with toxic lithium levels of 1.5 mmol/l, compared to 0.42 mmol/l in our case. Nowadays lithium levels are routinely checked in the last weeks before delivery, thus toxic lithium levels are rare. Whether this atrial flutter was a result of lithium toxicity or a congenital condition, is unclear. With no significant risk of neonatal complications in lithium-exposed newborns, our study, except for their finding of lower Apgar scores, is in accordance with a recent cohort study (Sagué-Vilavella et al. 2022). Obstetric vulnerability in our sample of women with BD was marked by higher levels of caesarean section (Seijmonsbergen-Schermers et al. 2018), gestational diabetes (Horsselenberg et al. 2021), and hypertensive disorders (Koopmans et al. 2009). This is in line with findings of various studies, who previously described high risk obstetric profiles of women with BD (Rusner et al. 2016; Frayne et al. 2018). Maternal obesity, high prenatal stress levels, smoking during pregnancy and comorbid psychiatric medication use may increase this vulnerability and alter birth outcomes (Rusner et al. 2016). In our sample, 11.1% of all women with BD smoked during all trimesters of pregnancy. Although the percentage of women smoking in the lithium and non-lithium exposed groups were comparable, smoking is a potential confounder with regards to neonatal morbidity. Preterm birth was not associated to lithium exposure in our study (p = 1.00), in contrast to previous research (Hastie et al. 2021). Although we did not find a difference in large for gestational age neonates, we found higher birth weight in lithium-exposed neonates and higher percentile birth weight compared to non-exposed neonates in line with previous studies (Poels et al. 2021).

## Conclusions

The results of this study show that one in five neonates was admitted to a neonatal ward with monitoring. Obstetric risks of mothers with BD were high and overall neonatal admissions were frequent. However, lithium exposure in itself was not a reason for admission to a neonatal ward with monitoring. We argue that special measures with regards to lithium use might be abundant, and advise joint observation of mothers with BD and their offspring in a nursery (level 1 care) to promote mother-infant bonding. Future studies should further explore factors related to the mental disorder in relation to obstetric vulnerability and adverse neonatal outcomes in women with BD.

## Data Availability

The datasets used and/or analysed during the current study are available from the corresponding author on reasonable request.
